# Effects of high-intensity interval training with hyperbaric oxygen

**DOI:** 10.3389/fphys.2022.963799

**Published:** 2022-08-19

**Authors:** Miguel Alvarez Villela, Sophia A. Dunworth, Bryan D. Kraft, Nicole P. Harlan, Michael J. Natoli, Hagir B. Suliman, Richard E. Moon

**Affiliations:** ^1^ Center for Hyperbaric Medicine and Environmental Physiology, Duke University Medical Center, Durham, NC, United States; ^2^ Department of Anesthesiology, Duke University Medical Center, Durham, NC, United States; ^3^ Department of Medicine, Division of Pulmonary, Allergy and Critical Care Medicine, Duke University Medical Center, Durham, NC, United States

**Keywords:** high-intensity interval training, hyperbaric oxygenation, mitochondrial turnover, high-altitude, oxygen consumption

## Abstract

Hyperbaric Oxygen (HBO_2_) has been proposed as a pre-conditioning method to enhance exercise performance. Most prior studies testing this effect have been limited by inadequate methodologies. Its potential efficacy and mechanism of action remain unknown. We hypothesized that HBO_2_ could enhance aerobic capacity by inducing mitochondrial biogenesis via redox signaling in skeletal muscle. HBO_2_ was administered in combination with high-intensity interval training (HIIT), a potent redox stimulus known to induce mitochondrial biogenesis. Aerobic capacity was tested during acute hypobaric hypoxia seeking to shift the limiting site of whole body V̇O2 from convection to diffusion, more closely isolating any effect of improved oxidative capacity. Healthy volunteers were screened with sea-level (SL) V̇O_2_peak testing. Seventeen subjects were enrolled (10 men, 7 women, ages 26.5±1.3 years, BMI 24.6±0.6 kg m^−2^, V̇O_2_peak SL = 43.4±2.1). Each completed 6 HIIT sessions over 2 weeks randomized to breathing normobaric air, “HIIT+Air” (PiO_2_ = 0.21 ATM) or HBO_2_ (PiO_2_ = 1.4 ATM) during training, “HIIT+HBO_2_” group. Training workloads were individualized based on V̇O_2_peak SL test. Vastus Lateralis (VL) muscle biopsies were performed before and after HIIT in both groups. Baseline and post-training V̇O_2_peak tests were conducted in a hypobaric chamber at PiO2 = 0.12 ATM. HIIT significantly increased V̇O_2_peak in both groups: HIIT+HBO_2_ 31.4±1.5 to 35.2±1.2 ml kg^−1^·min^−1^ and HIIT+Air 29.0±3.1 to 33.2±2.5 ml kg^−1^·min^−1^ (*p* = 0.005) without an additional effect of HBO_2_ (*p* = 0.9 for interaction of HIIT x HBO_2_). Subjects randomized to HIIT+HBO_2_ displayed higher skeletal muscle mRNA levels of *PPARGC1A*, a regulator of mitochondrial biogenesis, and *HK2* and *SLC2A4*, regulators of glucose utilization and storage. All other tested markers of mitochondrial biogenesis showed no additional effect of HBO_2_ to HIIT. When combined with HIIT, short-term modest HBO_2_ (1.4 ATA) has does not increase whole-body V̇O_2_peak during acute hypobaric hypoxia. (ClinicalTrials.gov Identifier: NCT02356900; https://clinicaltrials.gov/ct2/show/NCT02356900).

## Introduction

Hyperbaric oxygen (HBO_2_) has been proposed as a method for pre-conditioning to enhance exercise performance. Many athletes have admitted to using HBO_2_ (for instance, 1.3 - 2.8 atmospheres absolute [ATA] for 50 - 90 minutes) based on the principle that short exposures to HBO_2_ immediately before maximal exercise testing facilitates “oxygen loading” (Cabric et al., 1991; McGavock et al., 1999; Rozenek et al., 2007; Kawada et al., 2008). While tissue oxygen levels normalize almost immediately after removal from the hyperoxic environment, in one study, HBO_2_ therapy led to increased peak oxygen uptake (V̇O_2_peak) that was linked to increased skeletal muscle mitochondrial mass (Hadanny et al., 2022).

Hyperbaric oxygen has also been studied in combination with exercise training, albeit with mixed results. When administered in combination with moderate continuous training in athletes, it resulted in no additional improvements on V̇O_2_peak compared with training under normoxic conditions (Burgos et al., 2016). However, in a more recent study, HBO_2_ was administered in combination with high-intensity interval training (HIIT) and led to increased peak work rate and peak minute ventilation compared with air controls, but no differences in V̇O2peak (DeCato et al., 2019). The underlying hypothesis is that the mitochondrial bioenergetic program is synergistically activated by these two stimuli, potentially via redox signaling. The mitochondrial biogenesis program is cytoprotective and generates and distributes healthy mitochondria throughout the cell in response to oxidant and other stresses (Piantadosi and Suliman, 2012). Given the link between mitochondrial mass and aerobic capacity (Weibel et al., 1991), we proposed that induction of the mitochondrial biogenesis program would result in detectable improvements in V̇O_2_peak.

The co-activator and binding partner peroxisome proliferator-activated receptor gamma coactivator 1-alpha (PGC-1α) is a reliable marker for activation of this bigenomic program in human skeletal muscle. Both endurance and interval exercise training consistently increase its expression (Pilegaard et al., 2003; Calvo et al., 2008; Gibala et al., 2009; Olesen et al., 2010), and there is evidence that the relationship between PGC-1α and exercise may be bidirectional, as overexpression of this molecule in transgenic mice increases mitochondrial content and, in turn, improves exercise performance (Calvo et al., 2008). Activation of skeletal muscle PGC-1α is also seen with low-dose hyperbaric exposure (1.25 ATA with FiO_2_ 0.36) (Takemura and Ishihara, 2017) and other studies have found HBO_2_ can activate mitochondrial biogenesis in the brain (Gutsaeva et al., 2006; Hsu et al., 2022). Hence, HIIT and HBO_2_ together could synergistically increase PGC-1α and improve exercise capacity. However, HBO_2_ showed a neutral effect on V̇O_2_peak compared to HIIT alone (DeCato et al., 2019).

In the present study we directly examined the combined effect of HBO_2_ and HIIT on skeletal muscle bioenergetics as well as the attendant changes on V̇O_2_peak during acute hypobaric hypoxia. We chose a HIIT regimen similar to that employed by DeCato, et al. (DeCato et al., 2019), and measured changes in exercise capacity in a hypobaric hypoxic environment because hypoxic conditions (reduced oxygen supply) were believed to emphasize the role of peripheral determinants of V̇O_2_ (i.e. capillary surface area and mitochondrial capacity) more so than central determinants (i.e. lung capacity, heart function, and oxygen carrying capacity) (di Prampero, 2003). This strategy therefore allowed us focus as much as possible on the mitochondrial contributions of VO_2_ and concurrently measure changes in the skeletal muscle mitochondrial biogenesis program.

## Materials and methods

### Subjects and study enrollment

After institutional review board (IRB) approval and written informed consent, healthy non-smoker volunteers, ages 18 to 35 years, were screened. Subjects were excluded who had chronic comorbidities, pregnancy, sickle cell heterozygosity (African-American candidates were screened), physical deconditioning (sea-level V̇O_2_peak below 35 mL.kg^-1^.min^-1^ for men or 30 mL.kg^-1^.min^-1^ for women) (Gill et al., 2014; Bennett-Guerrero et al., 2017; Bennett-Guerrero et al., 2021), or were unable to provide written informed consent in English.

### Study design overview

**FIGURE 1:**
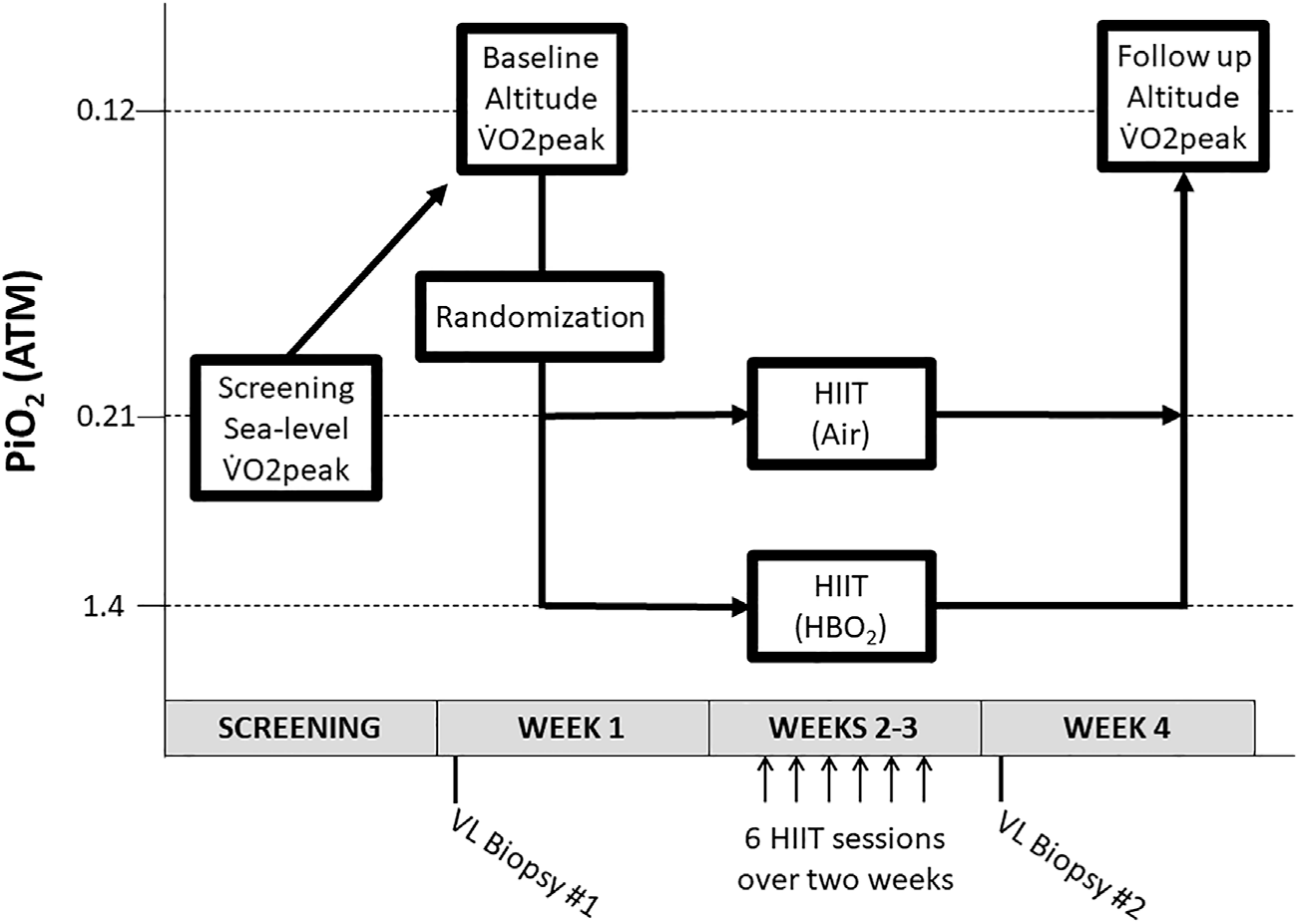
Study design. After a sea-level screening VO2peak test, subjects underwent a V̇O2peak test at altitude and were then randomized to HIIT + Air or HIIT + HBO_2_. After six HIIT sessions, subjects underwent a final V̇O2peak test at altitude. *Abbreviations*: ATM, atmospheres; HBO_2_, hyperbaric oxygen; HIIT, high-intensity interval training; PiO_2_, pressure of inspired oxygen; VL, vastus lateralis; V̇O2, oxygen consumption.

To determine study eligibility, potential subjects underwent a baseline medical history, physical exam, and sea-level (SL) graded maximal exercise test on a cycle ergometer. If eligible for participation, subjects were enrolled and underwent a vastus lateralis (VL) muscle biopsy followed 48 hours later by a graded maximal exercise test to exhaustion during acute hypobaric hypoxia (HH) in a hypobaric chamber decompressed to a barometric pressure of 429 mmHg (PiO_2_=0.12 atmospheres [ATM]) (Figure 1). Participants were then randomized 1:1 to a supervised, 6 session, high-intensity interval training program while breathing normobaric air, “HIIT+Air” group (PiO_2_=0.21 ATM), or 100% oxygen in a hyperbaric chamber “HIIT+HBO_2_” group (PiO_2_=1.4 ATM). Selection of the PO_2_ was based on a balance between the likelihood of a positive effect and an acceptable risk of central nervous system oxygen toxicity during exercise (Koch et al., 2013). One day after the last training session, a VL biopsy was obtained from the contralateral leg and a maximal exercise test during acute HH (PiO_2_=0.12 ATM) was repeated after a two-day recovery period. Subjects were instructed to continue their usual level of activity during the study, to avoid caffeine beverages on the day of exercise testing, and to avoid non-steroidal anti-inflammatory drugs (NSAIDs) 24 hours before muscle biopsies.

### Exercise protocol

All exercise sessions were performed on an upright cycle ergometer (Monark, model 818E). Seat height, handlebar elevation and angle and pedal strapping established during the initial briefing visit were maintained for each subject in all training and testing sessions.

Sea-level maximal exercise testing protocol: Subjects cycled at 75 rpm starting at 50 Watts (W) and workload was increased by 50 W increments every 3 minutes. Verbal encouragement was provided throughout the test to elicit maximal effort. Continuous ECG and pulse-oximetry monitoring were provided. The test was terminated for inability to maintain cadence, muscle cramping or subject request. During each test, subjects wore a nose-clip and breathed air through a two-way non-rebreathing valve (Hans Rudolf, model 2700) connected to a metabolic cart (Consentius Technologies, ParvoMedics TrueMax 2400). Mixed expired oxygen (O_2_), carbon dioxide (CO_2_), respiratory rate, minute ventilation, respiratory exchange ratio (RER), O_2_ consumption (V̇O_2_), and CO_2_ elimination (V̇CO_2_) were measured with the metabolic cart and recorded every 30 s as 30 s averages. V̇O_2_peak was recorded as the highest O_2_ uptake (V̇O_2_) reached during the test. Ventilatory threshold (VT) was calculated using the modified V-slope method (30s averages) (Gaskill et al., 2001).

**FIGURE 2:**
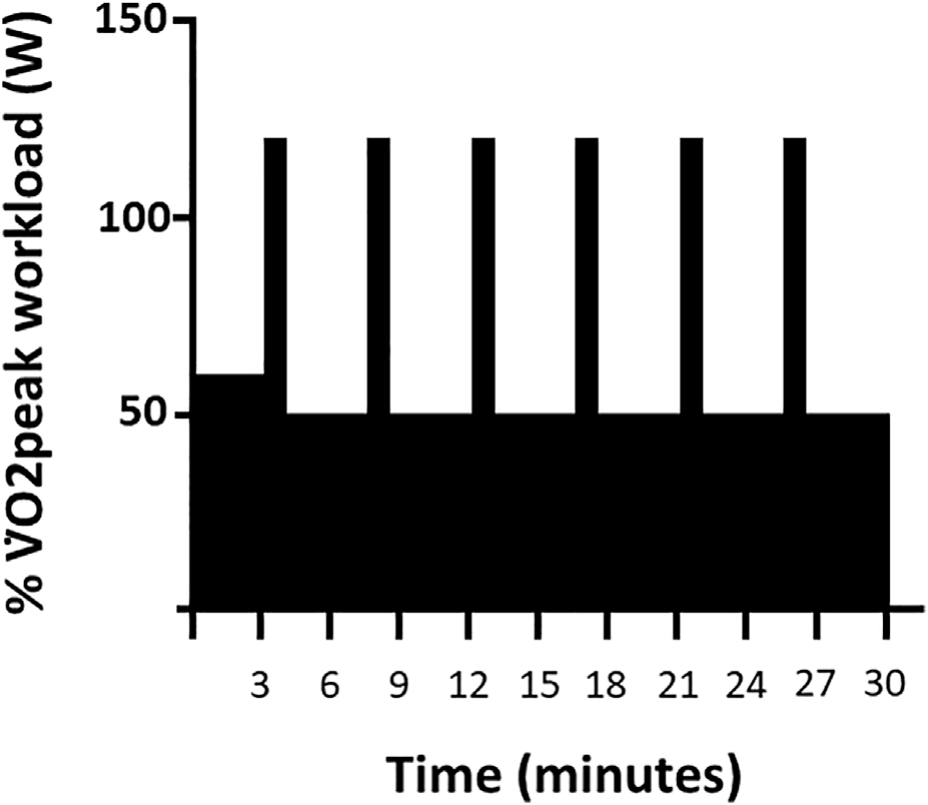
High-Intensity Interval Training. Timeline of a single 30-min HIIT session as a function of percent of V̇O2peak workload (W).

High Intensity Interval Training: Six training sessions were completed over a two-week period (Figure 1) by subjects in both groups using identical cycle ergometer setups and two-way non-rebreathing valve systems. Continuous ECG and periodic blood pressure monitoring were performed during each session. Subjects in the HIIT+Air group acted as controls training in our physiology laboratory at sea-level with the on-demand breathing system in direct communication with surrounding ambient air. Subjects randomized to train in the HBO_2_ group were pressurized in a dry hyperbaric chamber to a pressure of 1.4 ATA over 1 minute, or slower depending upon subject comfort. The described on-demand breathing system was connected to a pure oxygen source (FiO_2_=100%). A study investigator acted as a tender inside the chamber throughout each training session. Subjects were attached to a safety harness to avoid trauma in the event of an oxygen-induced convulsion. The chamber was decompressed back to sea-level pressure (1 ATA) over 2 minutes. Each training session consisted of a total of 30 minutes of exercise: Three minutes of warm-up, followed by six 30-second high-intensity interval periods interspersed with six 4-minute active recovery periods (Figure 2). Prescribed workloads for each period were calculated as percentages of the subject’s own peak workload (Watts) achieved during the screening SL maximal exercise test: 60% of peak output for the warm-up period, 120% for the high-intensity intervals and 50% for the recovery periods (Figure 2). During training, subjects were encouraged to maintain a cadence of 75 rpm while the pedaling resistance was adjusted by the supervising investigator to the prescribed workload for each period.

Acute hypobaric hypoxia maximal exercise testing: Maximal exercise tests were performed 1 week before and 1 week after the last HIIT session (Figure 1) in a hypobaric chamber at a simulated atmospheric pressure of 429 mmHg and FiO_2_=21%, (PiO_2_=0.12 ATM). Workload increments at altitude were 25W every 3 minutes. For each test, the chamber was depressurized at 2,000 ft per minute for 7.5 minutes to 15,000 ft. Recalibration of the metabolic cart was performed at target atmospheric pressure. A study investigator was present inside the chamber throughout completion of the test and the subject was attached to a safety harness to avoid trauma in the event of hypoxia-induced syncope. In addition to inability to maintain cadence, subject request and muscle cramping, tests were also terminated for severe hypoxemia (persistent O_2_ saturation of <70% on pulse-oximetry).

### Skeletal muscle biopsies

Vastus lateralis (VL) muscle biopsies were performed under sterile technique, using local anesthesia and a University College Hospital Muscle Biopsy Needle (120 mm, 5.0 mm OD, Popper & Sons, New Hyde Park, NY). Samples were obtained (total weight 200 mg), snap frozen in liquid nitrogen, and stored at -80°C.

### Western blot studies

Muscle proteins were separated by SDS-PAGE and identified by Western blot analysis (Suliman et al., 2007a). Membranes were incubated with validated antibodies against mitofusin-2 (Mfn2, 1:800, sc-50331), dynamin-1-like protein (Drp1, 1:1000, sc-271583), citrate synthase (1:2000, ab129095), ATP synthase subunit a (ATPase6, 1:1000, ab219825), cytochrome c oxidase (COI, 1:1000, PA5-26688), NADH dehydrogenase subunit 1 (ND1, 1:1000, ab181848), catalase (1:1500, ab209211), heme oxygenase-1 (HO-1, 1:1000, BML-HC3001), and superoxide dismutase-2 (SOD2, 1:2000, sc-137254), using GAPDH (1:5,000, G9545) or porin (1:5,000, PC548) antibody (Sigma, St. Louis, MO) as a loading control. After three washes in Tris-buffered saline with Tween, membranes were incubated with the appropriate horseradish peroxidase-conjugated secondary antibody (anti-goat, sc-2354, or anti-rabbit sc-2357 antibodies, both 1:10,000). Blots were developed with enhanced chemiluminescence (Western Blotting Luminol Reagent, sc-2048). Proteins were quantified by densitometry on digitized images from the mid dynamic range using Quantity One (Bio-Rad) and expressed relative to GAPDH or porin.

### Polymerase chain reaction studies

Total RNA was extracted from the muscle samples and analyzed by RT-PCR as previously described (Pecorella et al., 2015). RNA (1 μg) was reverse-transcribed by using random hexamer primers and a Superscript enzyme (Invitrogen) and real-time PCR was performed with a StepOne plus and gene expression master mix (Applied Biosystems, Foster City, CA). Primers and probes from Applied Biosystems were used for nuclear respiratory factor-2 (NRF2), peroxisome proliferator-activated receptor gamma coactivator 1-alpha (PPARGC1A), mitochondrial transcription factor A (TFAM), DNA polymerase subunit gamma-1 (POLG), mitochondrial DNA-directed RNA polymerase (POLRMT), glucose transporter 4 (SLC2A4), glucose transporter 1 (SLC2A1), and hexokinase-2 (HK2). 18S rRNA was used as an internal control. For the quantitative RT-PCR (qPCR) reaction, a difference of 1.0 in Ct value represents a twofold difference in transcript level. QRT-PCR was performed in triplicate. Quantitative PCR was then used to measure mtDNA copy number. Total mtDNA was isolated from muscle samples and determined by real-time PCR as described (Pecorella et al., 2015). Briefly, RT-PCR was performed using SYBR green qPCR Super Mix UDG (Invitrogen). The StepOne plus sequence detector system (Applied Biosystems) was used to record and analyze fluorescence intensities during PCR.

### Statistical analyses

Based on previous studies by our laboratory measuring PGC-1α protein expression in healthy subjects after exercise training, we calculated that a sample size of at least 18 subjects (9 in each group) was required for 80% power to detect a significant change (alpha 0.05) in molecular markers of mitochondrial biogenesis following our intervention. The pre-specified primary outcomes included change (pre-HIIT vs. post-HIIT) in V̇O2peak, V̇O2 at VT, mitochondrial mass, Tfam expression, and PGC-1α expression. Grouped data are reported as means ± SEM unless otherwise indicated, and analyzed via repeated-measures two-way ANOVA (or if missing values, by mixed-effects analysis) with Fisher’s least significant difference post-hoc testing (GraphPad Prism v8, San Diego, CA). *p* <0.05 was considered statistically significant.

## Results

### Enrollment and baseline characteristics

Twenty-five subjects were screened, four were excluded prior to randomization: one for below cut-off sea-level V̇O_2_peak, one due to syncope during first VL biopsy, and two because of training session scheduling conflicts. Twenty-one subjects completed the study protocol. Four were excluded from the data analysis due to submaximal effort on sea-level or altitude testing (RER<1.0), or due to technical failures related to calorimetric gas analysis at altitude. Therefore, 17 subjects (10 men, 7 women) were included in the data analysis. Nine were in the air group and 8 in the HBO_2_ group. Ages ranged 19–35 years, BMI 24.6±0.6 kg m^−2^, sea-level (SL) V̇O_2_peak 43.4±9 ml kg^−1^·min^−1^and baseline hypobaric hypoxia (HH) V̇O_2_peak 30.1±1.7 ml kg^−1^·min^−1^ (Table 1). Baseline characteristics are summarized in Table 1. Subjects in both groups were similar in age, BMI and fitness level (V̇O_2_peak SL HIIT+HBO_2_ group = 45.2±2.69 ml·kg·min^−1^ vs. HIIT+Air 41.7±3.30 ml kg^−1^·min^−1^; *p* = 0.31).

**TABLE 1:** Baseline Subject Characteristics including all subjects. All measurements performed before training at sea-level (SL) and during hypobaric hypoxia (HH) (mean ± SEM). †*p*-value represents comparison between groups. HH testing performed in a hypobaric chamber, PiO_2_ = 0.14. *Abbreviations*: BMI, body mass index; HR, heart rate; PiO_2_, pressure of inspired oxygen; RER, respiratory exchange ratio; SBP, systolic blood pressure; SL, sea-level; V̇O_2_peak, peak oxygen consumption.

	HIIT+Air	HIIT+HBO_2_	All	*p*-Value†
N	9	8	17	
Age (years)	27.8 ± 1.9	25.0 ± 1.7	26.5 ± 1.3	0.3
Sex (M/F)	5/4	5/3	10/7	
BMI (kg/m2)	25.4 ± 0.8	23.7 ± 0.8	24.6 ± 0.6	0.14
Body Fat (%)	19.1 ± 3.1	13.8 ± 2.3	16.6 ± 2.0	0.18
Resting HR SL (bpm)	76.9 ± 4.3	72.0 ± 3.2	74.6 ± 2.7	0.37
Resting SBP SL (mmHg)	121.2 ± 3.7	118.6 ± 4.2	120.1 ± 2.7	0.64
V̇O_2_peak SL (mL·kg^−1^·min^−1^)	41.7 ± 3.3	45.2 ± 2.7	43.4 ± 2.1	0.42
RER SL	1.21 ± 0.03	1.24 ± 0.01	1.23 ± 0.02	0.3
Peak Work SL (Watt)	253.9 ± 18.4	237.4 ± 22.0	246.2 ± 13.9	0.57
Baseline V̇O_2_peak HH (mL·kg^−1^·min^−1^)	29.0 ± 3.1	31.4 ± 1.5	30.1 ± 1.7	0.49
RER HH	1.32 ± 0.07	1.34 ± 0.06	1.33 ± 0.05	0.84

### Effects of acute hypobaric hypoxia

Maximal exercise testing during acute HH (barometric pressure = 429mmHg; PiO_2_ = 0.12 ATM) resulted in an equal (non-statistically significant) Δ V̇O_2_peak from sea-level in both groups, -13.5±4.6 ml kg^−1^·min^−1^ in the HIIT+HBO_2_ group (48% decrease from baseline) and -13±3.9 ml kg^−1^·min^−1^ in the HIIT+Air group (56% decrease from baseline). A linear correlation was observed between Δ V̇O_2_peak with acute altitude exposure and sea-level V̇O_2_peak values, with the largest drops occurring in the fittest subjects (R = 0.8; *p* <0.001). This was in keeping with prior reports on the effect of acute hypoxia upon peak oxygen consumption (Lawler et al., 1988).

### Physiological effects of high-intensity interval training at sea-level or hyperoxic-hyperbaric environment

Heart rates were measured for each subject during the first, third and sixth recovery periods of the 1^st^ and 6^th^ training sessions but were not statistically significant between the two groups (152 ± 13 in the HIIT+Air group and 142 ± 21 in the HIIT+HBO_2_ group). Average workload (Watts) performed during each training session was also similar between both groups, 736±53.7 W in the HIIT+Air group and 688±64 W in the HIIT+HBO_2_ group (*p* = 0.4). Physiologic and hemodynamic parameters during maximal exercise testing before and after training are detailed in Table 2. The addition of HBO_2_ to HIIT did not result in an additional effect on V̇O_2_ at VT or during peak exercise compared to the effect of HIIT alone (*p* = 0.0045 for training effect, *p* = 0.9 for training x exposure effect). VT as V̇O_2_peak % remained unchanged in both groups after training (air, 73.2±2.5 to 70±2.0% and HBO_2_, 66.2±2.7 to 67±2.8%). There was no significant effect of training or HBO_2_ exposure on systolic blood pressure (SBP) or heart rate (HR) at rest and at peak exercise. There was, however, a significant effect of HBO_2_ on attenuating pulse pressure rise during exercise (*p* = 0.05 for interaction, post-hoc comparison).

**TABLE 2:** Physiologic effect of training in all subjects. N = 17. All measurements done during acute hypobaric hypoxia (HH), 429 mmHg barometric pressure (PiO_2_ = 0.14) before and after training (mean ± SEM). *p <0.05. *Abbreviations*: HH, hypobaric hypoxia; HR, heart rate; PP, pulse pressure; SBP, systolic blood pressure; V̇O_2_peak, peak oxygen uptake; VT, ventilatory threshold by modified V-slope method.

	HIIT+Air		HIIT+HBO_2_		*p*-Value Training Effect	*p*-Value Interaction Training x Exposure
	Pre	Post	Pre	Post		
V̇O_2_peak HH (mL·kg^−1^·min^−1^)	29.0 ± 3.1	33.2 ± 2.5	31.4 ± 1.5	35.2 ± 1.2	0.0045*	0.9
VT HH (mL·kg^−1^·min^−1^)	21.3 ± 2.4	23.6 ± 2.0	18.6 ± 1.8	21.8 ± 2.0	0.016*	0.7
Peak Workload HH (W)	214 ± 13	217 ± 22	191 ± 13	200 ± 16	0.32	0.6
Resting SBP HH (mmHg)	118 ± 4	120 ± 4	125 ± 7	117 ± 4	0.14	0.6
Resting HR HH (bpm)	95 ± 3	92 ± 2	98 ± 9	88 ± 6	0.15	0.3
Peak SBP HH (mmHg)	148 ± 5	154 ± 6	141 ± 7	139 ± 7	0.8	0.4
Peak HR HH (bpm)	178 ± 5	176 ± 4	174 ± 3	178 ± 3	0.6	0.6
Increase in PP Rest to Peak HH Exercise (mmHg)	29	41	28	23	0.7	0.05

### Safety of training and hypobaric and hyperbaric exposures

No adverse events occurred during training. In the HBO_2_ group, there were no complications related to chamber compression/decompression and no oxygen-induced convulsions or symptoms suggestive of oxygen toxicity, such as seizures or reductions in forced vital capacity. All acute high-altitude exposures were well tolerated, with no syncopal or pre-syncopal events. No tests were stopped due to hypoxemia (defined as SpO_2_<70%). One subject suffered vasovagal syncope during baseline muscle biopsy with uneventful recovery and one other subject declined repeat biopsy due to discomfort.

### Effects of high-intensity interval training and hyperbaric oxygen on skeletal muscle

**FIGURE 3:**
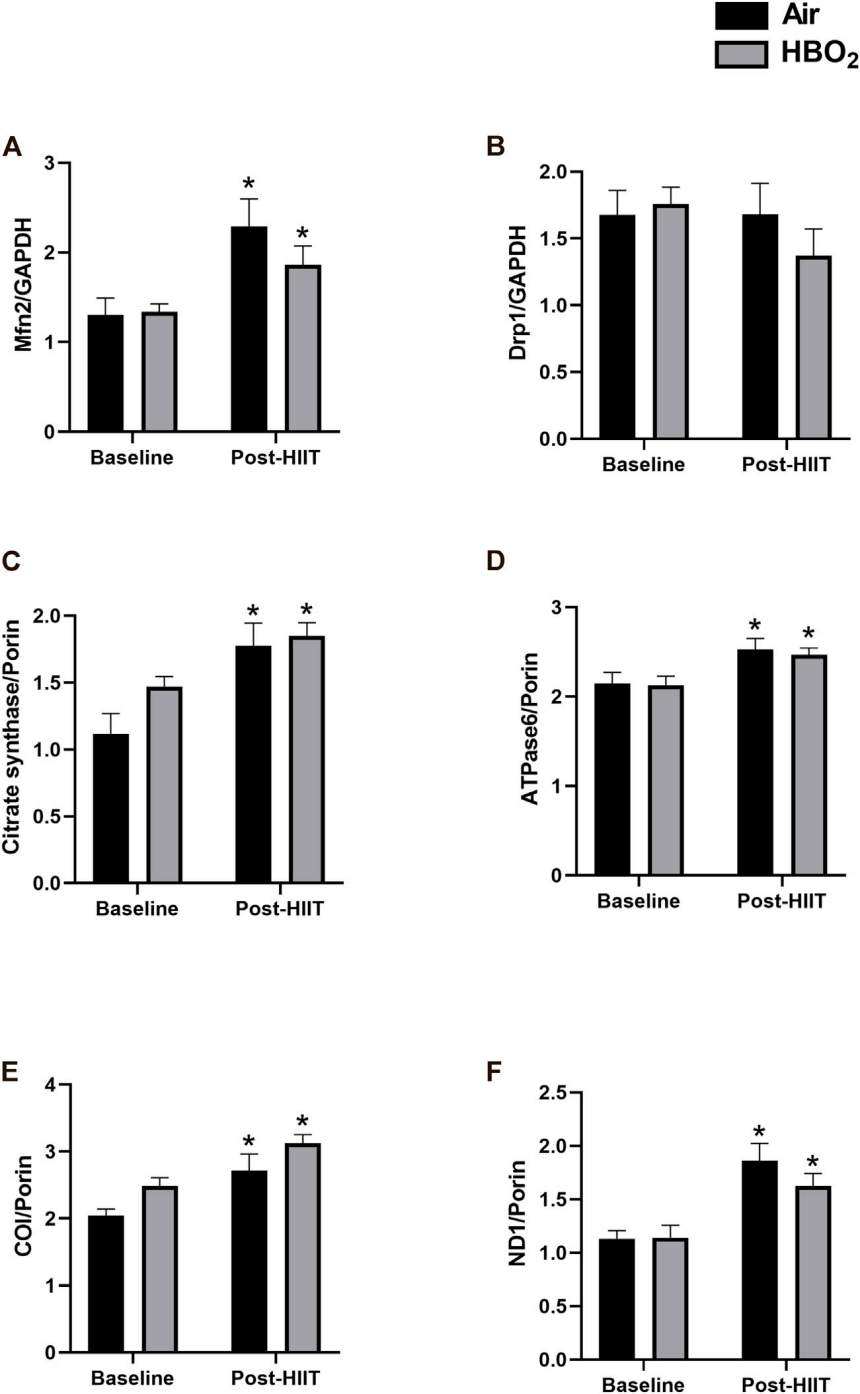
Mitochondrial protein expression. Expression of the following skeletal muscle proteins was measured by western blot: **(A)** Mfn2, mitofusin-2; **(B)** Drp1, dynamin-1-like protein; **(C)** Citrate synthase; **(D)** ATPase6, mitochondrial ATP synthase subunit a; **(E)** COI, cytochrome c oxidase subunit 1; and **(F)** ND1, NADH-ubiquinone oxidoreductase chain 1. Reference proteins were either GAPDH or Porin. Statistical analysis was 2-way ANOVA with repeated measures and Fisher’s LSD post-hoc test. *p <0.05 shows significant within group comparison (i.e. baseline vs. post-HITT). Groups: air, black bars; HBO_2_, grey bars.

**FIGURE 4:**
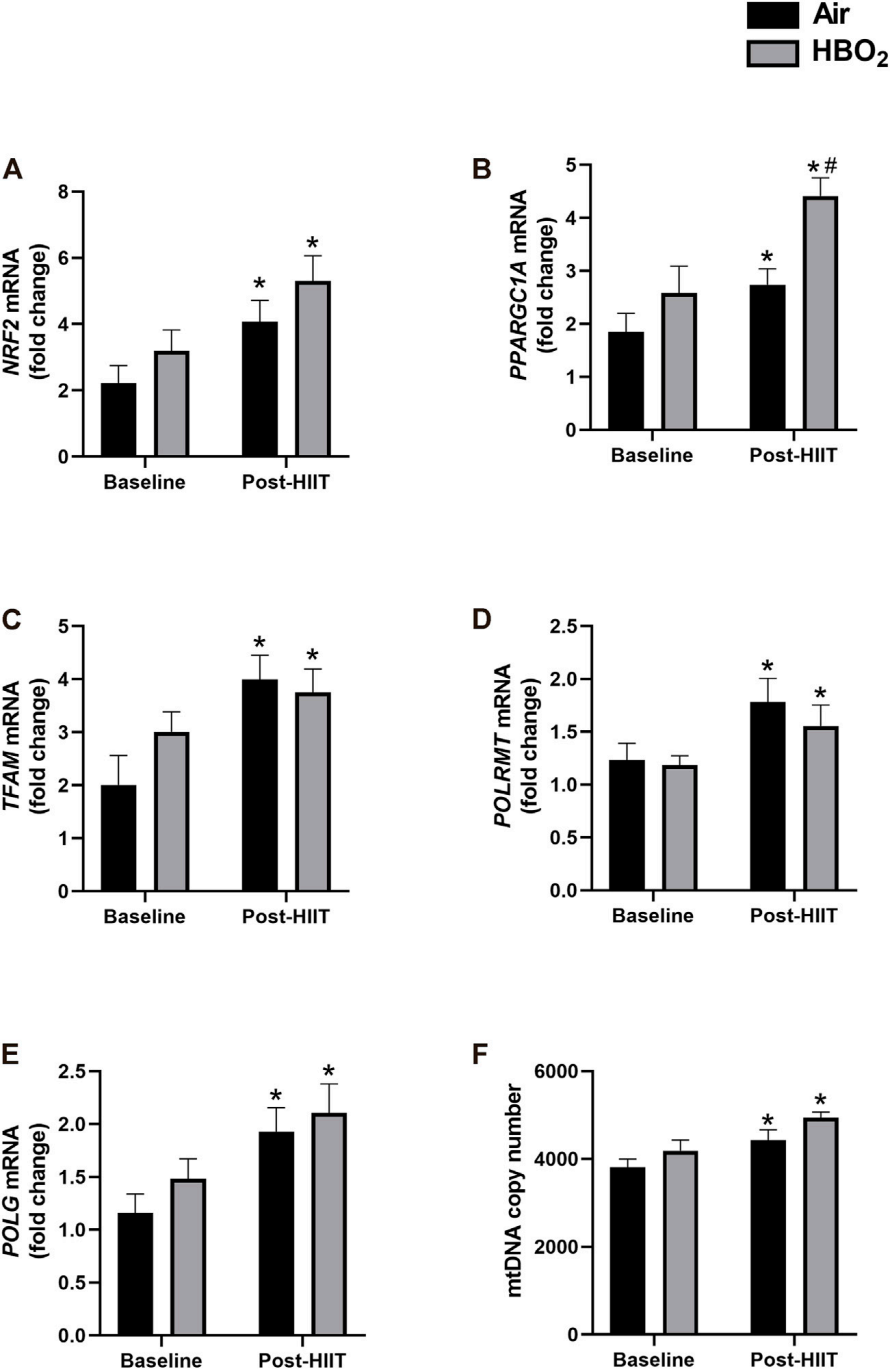
Mitochondrial gene expression. mRNA levels of the following genes were measured by quantitative PCR: **(A)** *NRF2*, nuclear respiratory factor 2; **(B)** *PPARGC1A*, peroxisome proliferator-activated receptor gamma coactivator 1-alpha; **(C)** *TFAM*, mitochondrial transcription factor a; **(D)** *POLG*, DNA polymerase subunit gamma-1; and **(E)** *POLMRT*, mitochondrial DNA-directed RNA polymerase. Reference mRNA was 18S and shown as fold-change. Mitochondrial DNA copy number was measured in **(F)** by PCR with 18S rDNA as reference and shown as fold-change. Statistical analysis was 2-way ANOVA with repeated measures and Fisher’s LSD post-hoc test. *p <0.05 shows significant within group comparison (i.e. baseline vs. post-HITT) and ^#^ *p* <0.05 shows significant between group comparisons (i.e. air, black bars vs. HBO_2_, grey bars).

Skeletal muscle biopsies from a subset of subjects (HIIT+Air, n = 7; HIIT+HBO_2_, n = 6) were analyzed for changes in protein and gene expression. We found increased protein expression in mitofusin-2 (Mfn2), citrate synthase, ATPase 6, cytochrome c oxidase (COI), and ND1 from exercise training alone, but the addition of HBO_2_ had no effect (Figure 3). Neither intervention altered expression of Drp1. Quantitative PCR analysis showed significantly increased muscle expression of *NRF2* and *TFAM*, two genes critical to regulation of mitochondrial biogenesis, from exercise training alone but not from HBO_2_ (Figure 4). We also found that exercise training but not HBO_2_ increased gene expression of the polymerases *POLG* and *POLRMT*, which are necessary for mitochondrial DNA replication and transcription, respectively. Moreover, compared to baseline values, there was significantly higher *PPARGC1A* expression and mtDNA copy number in muscle samples taken after training. However, with HIIT+HBO_2_, *PPARGC1A* expression was further augmented compared with the HIIT+Air group (*p* = 0.005).

**FIGURE 5:**
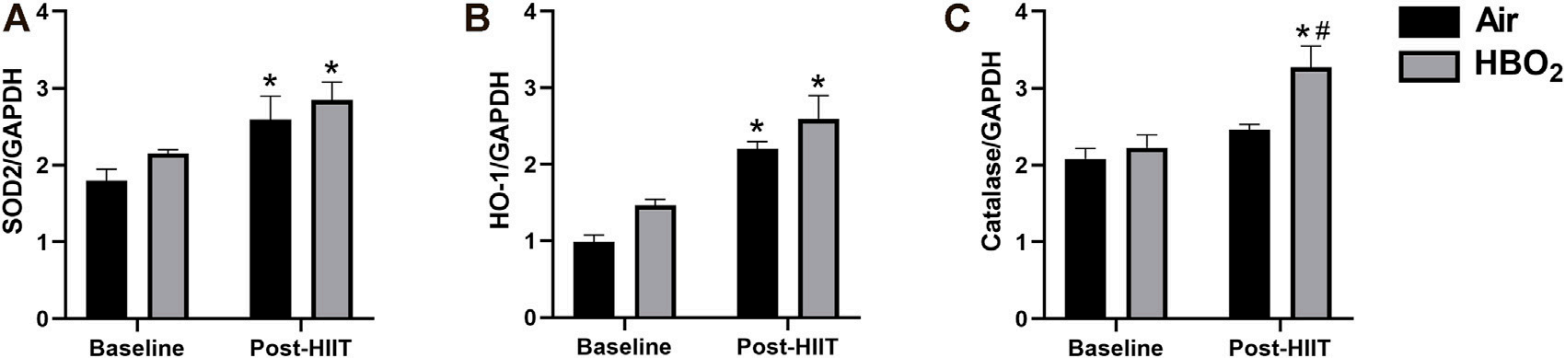
Antioxidant response. Antioxidant protein expression of **(A)** SOD2, superoxide dismutase-2; **(B)** HO-1, heme oxygenase-1; and **(C)** catalase were measured by western blot and referenced to GAPDH expression. Statistical analysis was 2-way ANOVA with repeated measures and Fisher’s LSD post-hoc test. *p <0.05 shows significant within group comparison (i.e. baseline vs. post-HITT) and ^#^ *p* <0.05 shows significant between group comparisons (i.e. air, black bars vs. HBO_2_, grey bars).

Because both HIIT and HBO_2_ stimulate ROS production that may activate anti-oxidant responses, we measured expression of select mitochondrial anti-oxidant proteins (Figure 5). We found significantly increased expression of HO-1 and SOD2 with exercise training in both groups but no effect from HBO_2_. However, compared to baseline and to the HIIT+Air Group, the addition of HBO_2_ to HIIT significantly increased skeletal muscle catalase expression (*p* <0.005).

**FIGURE 6:**
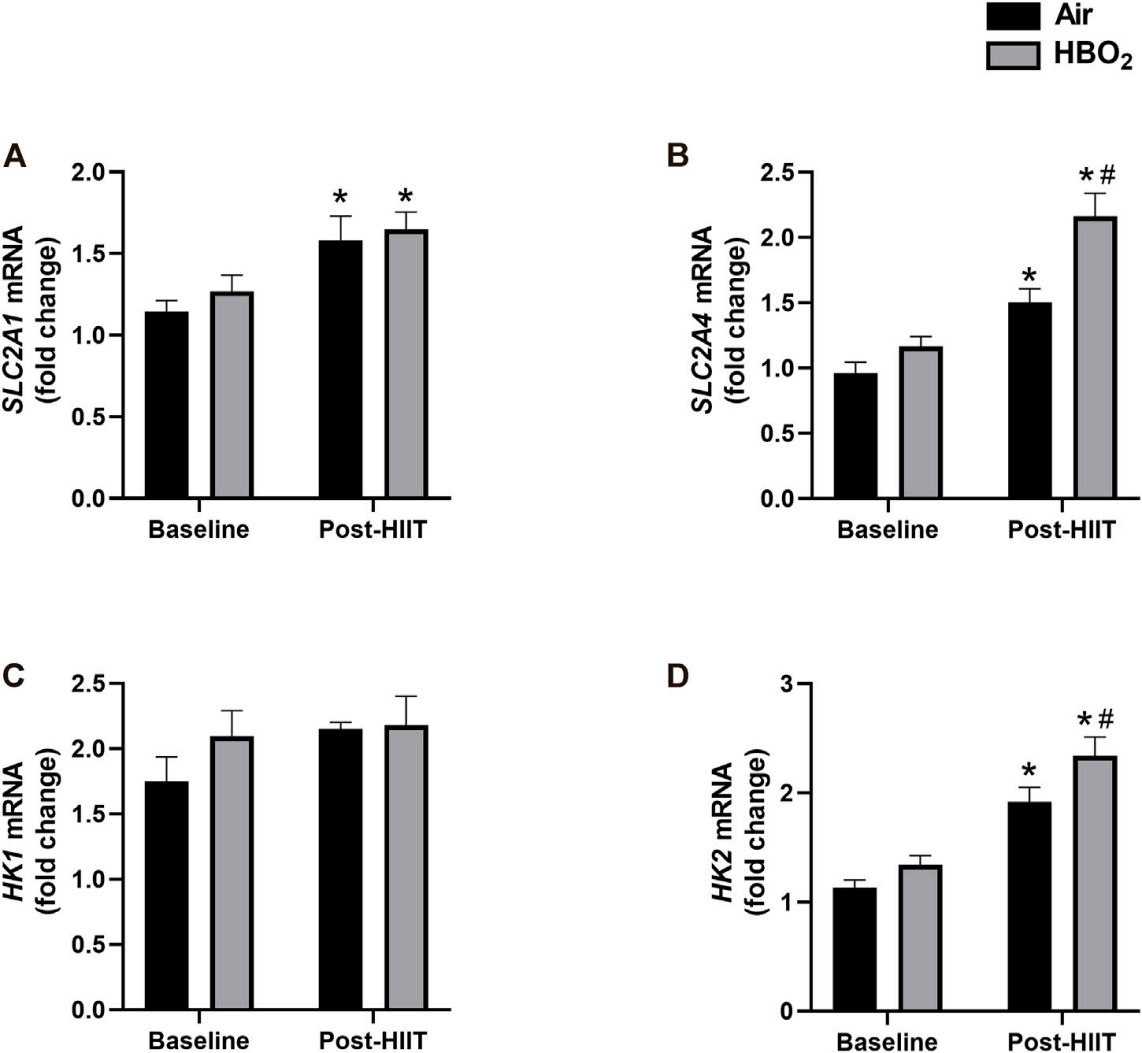
Glucose-related gene expression. mRNA levels of the following genes were measured by quantitative PCR: **(A)** *SLC2A1*, Solute carrier family 2, facilitated glucose transporter member 1; **(B)** *SLC2A4*, Solute carrier family 2, facilitated glucose transporter member 4; **(C)** *HK1*, hexokinase-1; and **(D)** *HK2*, hexokinase-2. Reference mRNA was 18S and shown as fold-change. Statistical analysis was 2-way ANOVA with repeated measures and Fisher’s LSD post-hoc test. *p <0.05 shows significant within group comparison (i.e. baseline vs. post-HITT) and ^#^p <0.05 shows significant between group comparisons (i.e. air, black bars vs. HBO2, grey bars).

Finally, we investigated whether there were changes in markers of substrate (glucose) utilization (Figure 6). We found that exercise training alone significantly increased gene expression of the glucose transporters *SLC2A1* and *SLC2A4*; however, compared with air treated subjects, the addition of HBO_2_ further increased *SLC2A4* expression (*p* = 0.0007). Additionally, while neither HIIT nor HBO_2_ altered *HK1* expression, exercise training significantly increased *HK2* expression that was further increased by addition of HBO_2_ (*p* = 0.02).

## Discussion

The aim of our study was to examine the effect of HBO_2_ combined with HIIT on activation of mitochondrial biogenesis in skeletal muscle and maximal oxygen uptake during acute hypobaric hypoxia. Hypoxic conditions were implemented in an attempt to shift the whole-body oxygen uptake kinetics towards diffusion and extraction (greater dependence on mitochondrial function), and away from convection (di Prampero, 2003). HBO_2_ was administered in conjunction with HIIT, a known stimulator of mitochondrial biogenesis (Gibala et al., 2009; Little et al., 2010) and highly efficient method for improving aerobic capacity in trained and untrained individuals (Laursen and Jenkins, 2002; Hazell et al., 2010). Our findings found HIIT to be highly efficacious at improving V̇O_2_peak under acute hypoxic conditions. However, we found no added effect of HBO_2_ on aerobic capacity.

We also found no effect of HBO_2_ on the regulation of mitochondrial biogenesis compared with air controls. While *PPARGC1A* mRNA levels were significantly higher in subjects that trained with HBO_2_ compared with the air-only group (a primary endpoint, and to which our study was powered), the HBO_2_ group did not see increases in other associated markers, such as citrate synthase and ATPase6, which are measures of mitochondrial volume density, or *NRF2* and *TFAM* expression, which are measures of mitochondrial biogenesis activation. The reasons for this are not clear, but could possibly relate to differences in peak expression after HBO_2_, insufficient dose of HBO_2_ to fully activate mitochondrial biogenesis, or lack of biological effect by HBO_2_. These findings contrast with recent work by Hadanny, et al. that found increased V̇O2peak in healthy master athletes after HBO_2_ treatment that was linked to increased skeletal muscle mitochondrial mass (Hadanny et al., 2022). However, we are skeptical of these molecular findings, as MitoTracker Green fluorescence was measured in fixed tissues, but is only validated and specific in live cells, and the PG1-alpha protein measurements were not stated as the nuclear (activated) fraction. Moreover, no changes were seen in MFN1/2 or OPA1, which are markers for mitochondrial quality and integrity. Our study also differed from the Hadanny study, as it included younger subjects, fewer HBO_2_ sessions, lower doses of HBO_2_, and added the HIIT intervention. This latter difference may be most relevant, as we found mitochondrial biogenesis (using appropriate techniques) was activated by HIIT alone, leading to increased mitochondrial mass. In fact, the mitochondrial biogenesis program may have been maximally activated by HIIT and may have reduced our ability to detect further effects from HBO_2_. HIIT alone activated mitochondrial biogenesis (evidenced by increased *TFAM*, *NRF1*, and *PPARGCA1* gene expression), increased mitochondrial quality and integrity (increased MFN2 protein expression), and increased mitochondrial mass (increased citrate synthase and ATPase 6 protein expression). Furthermore, to our knowledge, this is the first study to translate from rodents to humans that interval exercise training increases gene expression of *POLG*, the mitochondrial DNA polymerase that repairs mtDNA damage (Clark-Matott et al., 2015) postulated to explain some of the health benefits of regular exercise (Garatachea et al., 2015). We also report for the first time in any species the exercise-induced gene expression of mitochondrial DNA-directed RNA polymerase (*POLRMT*), which transcribes mtDNA to RNA, and may be a novel biomarker for mitochondrial exercise response.

Breathing hyperbaric oxygen significantly raises tissue oxygen levels and increases production of reactive oxygen species (ROS), predominantly at the mitochondrial electron transport chain, where molecular oxygen is reduced to form superoxide (Jamieson et al., 1986). Superoxide is then converted by superoxide dismutase (SOD) to hydrogen peroxide (H_2_O_2_), a less toxic intermediate that is converted to water by catalase. We found that exercise but not HBO_2_ significantly increased mitochondrial SOD (SOD2) expression. However, only exercise and HBO_2_ together increased catalase expression. We are not sure why attendant increases in SOD2 after HBO_2_ were not seen, as HBO_2_ does increase superoxide formation (Jamieson et al., 1986; Groger et al., 2009), but possible explanations include missed peak expression, or reduced signal due to the highly efficient nature of the SOD system. It is also possible that the HBO_2_ host response is more dependent on cytosolic SOD3, which we did not measure. Regardless, the higher catalase expression seen after training with HBO_2_ likely reflects elevated tissue levels of H_2_O_2_. Hydrogen peroxide can activate PGC-1α expression (St-Pierre et al., 2006) as well as other cellular redox sensors (e.g. Akt) (Suliman et al., 2007b; Piantadosi and Suliman, 2012), providing other plausible mechanisms for the higher *PPARGC1* mRNA levels seen after HBO_2_.

Glucose is a critical energy source during exercise, and its metabolism is regulated by insulin-dependent cellular uptake via glucose transporters and step-wise enzymatic catabolism. We found evidence this system was upregulated by exercise, consistent with prior studies (Pilegaard et al., 2003; Little et al., 2010), and perhaps further augmented by addition of HBO_2_. Specifically, compared to sea-level training, HIIT+HBO_2_ subjects displayed significantly higher gene expression of *SLC2A4* (*GLUT4*), the dominant glucose transporter for skeletal muscle, and hexokinase-2 (*HK2*), a rate-limiting enzyme in glycolysis that also couples glucose metabolism to oxidative phosphorylation (Roberts and Miyamoto, 2015). These changes could be consistent with improved muscle bioenergetic efficiency, and if so, parallel those seen with exogenous CO administration (Rhodes et al., 2009).

Our study was limited by a number of factors. First, it is possible that maximal exercise testing is not the best test to examine the effects of changes in mitochondrial function, even under hypoxic conditions. Exercise efficiency during steady-state exercise or time to exhaustion during constant speed exertion have been proposed as more accurate methods for this purpose, and should be considered in future studies (Broskey et al., 2015). Secondly, our study design was based on the assumption that the rate-limiting step in V̇O_2_peak in healthy humans is oxygen supply. This was based on the concept of symmorphosis, which stipulates that biological structure is matched to physiological functional capacity. In the case of oxygen transport (except for lung capacity which exceeds demand), symmorphosis predicts matching of oxygen supply and demand in peripheral tissues at each step of the oxygen cascade (Weibel et al., 1991). Hence, we proposed that an acute reduction in ambient oxygen tension during maximal exercise would allow for a more accurate assessment of peripheral V̇O_2_ components, and more specifically, of mitochondrial oxidative capacity upon oxygen consumption rate (di Prampero, 2003). However, recent data calls into question the validity of symmorphosis in trained individuals, finding that V̇O_2_peak is limited by mitochondrial O_2_ demand rather than supply (Gifford et al., 2016). However, that study was conducted under different conditions (normoxia) and in different participants (endurance-trained athletes) than our study. Nevertheless, it is possible that the use of acute hypobaric hypoxia in our study to isolate mitochondrial O_2_ demand as a determinant of V̇O_2_peak may not have worked as expected. Third, it is possible that subjects in the HBO_2_ group trained at workloads that, although equivalent at sea-level to the Air group, represented a lower level of effort under their hyperoxic training condition. Rate of perceived exertion during training was not captured in our study. Fourth, the lack of morphological analysis of our muscle biopsies did not allow us to assess the effect of our intervention upon capillary density and muscle fiber remodeling, also important factors affecting the diffusion component of whole-body oxygen uptake. Fifth, we did not measure mitochondrial function (due to logistical and technical considerations) which may have uncovered underlying differences between the groups that were not otherwise evident. Finally, we cannot exclude the possibility of a beneficial training effect of higher PO_2_ (e.g. 2 ATA) when combined with HIIT; however, this was not studied to the greater risk of central nervous system oxygen toxicity.

In conclusion, our study found no beneficial effects of adding modestly-dosed HBO_2_ to HIIT for improving aerobic fitness and no definite molecular evidence of activation of skeletal muscle mitochondrial biogenesis. Future studies could investigate whether similar HBO_2_ doses improve glucose utilization and storage after HIIT and whether these accelerate recovery.

## Data Availability

The raw data supporting the conclusion of this article will be made available by the authors, without undue reservation.
